# Extraction of Betulin, Trimyristin, Eugenol and Carnosic Acid Using Water-Organic Solvent Mixtures

**DOI:** 10.3390/molecules17089274

**Published:** 2012-08-03

**Authors:** Fulgentius N. Lugemwa

**Affiliations:** Department of Chemistry, Pennsylvania State University-York, 1031 Edgecomb Avenue, York 17403, PA, USA; Email: ful4@psu.edu; Tel.: +1-717-771-8409; Fax: +1-717-771-4062

**Keywords:** toxic chemicals, liquid extraction, spices, antioxidants

## Abstract

A solvent system consisting of ethyl acetate, ethyl alcohol and water, in the volume ratio of 4.5:4.5:1, was developed and used to extract, at room temperature, betulin from white birch bark and antioxidants from spices (rosemary, thyme, sage, and oregano) and white oak chips. In addition, under reflux conditions, trimyristin was extracted from nutmeg using the same solvent system, and eugenol from olives was extracted using a mixture of salt water and ethyl acetate. The protocol demonstrates the use of water in organic solvents to extract natural products from plants. Measurement of the free-radical scavenging activity using by 2,2-diphenyl-1-picrylhydrazyl (DPPH) indicated that the extraction of plant material using ethyl acetate, ethyl alcohol and water (4.5:4.5:1, v/v/v) was exhaustive when carried out at room temperature for 96 h.

## 1. Introduction

Liquid extraction constitutes a critical step in the isolation of different compounds from terrestrial plants, marine organisms, and cultured broth. The pure compounds isolated from these sources can be used in basic or clinical research. Crude extracts obtained by liquid extraction are often used in cosmetics and food supplement industries or in pest management programs, where their uses are not regulated by the Food and Drug Administration. Solvent extraction has been carried out under different conditions. The raw material can be macerated and suspended in a solvent at room temperature for a few h to several days [1,2]; it can be boiled in organic solvents such as toluene [3]; or, can be directly extracted using a Soxhlet apparatus [4]. After removing the extracting solvent, pure compounds from crude extracts are obtained using different techniques that include various chromatographic methods, followed by crystallization, if the compounds are solids. Toxic organic solvents such as dichloromethane, chloroform, benzene, and toluene are frequently used for extracting compounds from different sources in natural products research [3,5,6,7,8,9,10]. After extraction, these solvents are easily removed under reduced pressure. Sometimes, preferential extraction of the compound of interest can be achieved if a proper solvent is used.

Hot water is also capable of extracting some polar organic compounds from natural products, for example, caffeine, polyphenols and other organic compounds can be extracted from tea and coffee. During the maturation of distilled spirits in wood casks, the ethyl alcohol-water mixture extracts several compounds from the wood during the slow maturation process [11,12]. The wood components that are hydrophobic and have low solubility in aqueous solvent systems include ellagic acid, furfural, vanillin, and syringaldehyde [13,14]. These extractions, though not efficient, demonstrate a possibility of using polar solvents to extract non-polar organic compounds, if the extraction conditions are optimized. Such efforts could lead to cheap and more eco-friendly processes that could be applied to various extraction processes, provided the compounds to be extracted are stable under those conditions. In this report, the extraction of organic compounds from plants using a mixture of water, ethyl acetate and ethyl alcohol is reported.

## 2. Results and Discussion

**Figure 1:**
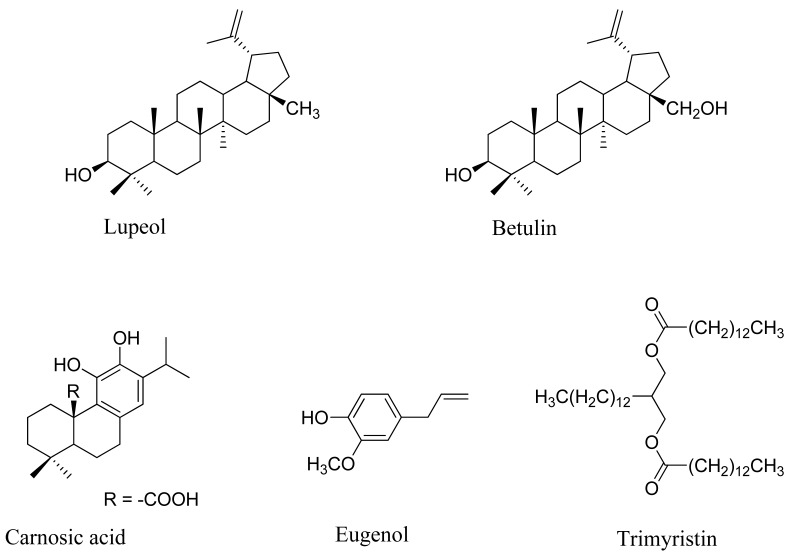
Compounds extracted using a mixture of water, ethyl acetate end ethyl alcohol.

Several bioactive compounds have been derived from betulin, but it is still possible to obtain many other derivatives through rearrangements, ring expansions, ring contractions, and cleavages that provide entry into the triterpene’s skeleton to carry out further functionalization. The potential of obtaining new model compounds makes it necessary to come up with new efficient and eco-friendly methods of obtaining large quantities of betulin from sustainable sources. The extraction of betulin from white birch bark has been carried out using different solvents [3,15,16]. The skeleton of betulin with the exception of two hydroxyl groups, located far apart at both ends of the molecule (Figure 1), is quite hydrophobic; however, betulin is soluble in acetic acid. We reasoned that a polar solvent system could be used to extract betulin at room temperature, even though previous extractions had been carried out successfully using relatively non-polar solvents like chloroform, dichloromethane and toluene under reflux conditions. In general, the extraction process is affected by solvent polarity, analyte polarity, temperature, and the extraction time. The solvent system consisting of ethyl acetate, ethyl alcohol, and water in the volume ratio of 4.5:4.5:1 was found to be the most efficient, and was obtained by a trial and error method. Extractions were carried out for 12, 24, 48 and 96 h at room temperature (data not shown). Exhaustive extraction was achieved after 96 h. Mixtures of ethyl acetate, ethanol, and water in other volume ratios were less effective in extracting betulin from white birch bark. The amount of water used could not exceed 10% because of miscibility problems with the organic solvents. Upon concentrating under vacuum, after extracting the plant material, the crude solid precipitated, as the amount of ethyl acetate and ethanol decreased, which simplified the isolation of the product. More ethyl acetate was added to the aqueous mixture after concentration, the organic layer separated and concentrated to produce a cleaner product that was devoid of the more polar water-soluble components. The total extract consisted of betulin, and lupeol as minor product. The presence of the two compounds was detected by thin layer chromatography using previously isolated standards. The recovery obtained after extracting with ethyl acetate and ethanol in the volume ratio of 1:1 was as good the one obtained by using ethyl acetate, ethanol, and water in the volume ratio of 4.5:4.5:1; extracting with either ethyl acetate or ethyl alcohol alone was less efficient (Table 1). Pure betulin from crude extracts was isolated after running a short silica gel column eluting with a mixture of hexane and ethyl acetate, followed by recrystallization from 75% ethyl alcohol (Table 2). After the first extraction, the second extraction of the plant material produced a total mass of material that was less than 1% of the first extract, indicating that the first extraction removed the majority of the compound. The amount of pure betulin obtained by using ethyl acetate, ethyl alcohol and water was the same as that obtained by using hot toluene. The identity of betulin and lupeol were confirmed using ^1^H-NMR.

**Table 1:** Total extract from 20 g white birch bark.

Solvent system	Extract (g)
Ethyl acetate:ethanol:water (4.5:4.5:1,v/v/v)	4.4
Ethyl acetate and ethanol (1:1,v/v)	4.5
Ethanol	2.2
Ethyl acetate	2.5
Ethyl acetate:ethanol:water (6:3:1)	3.7
Ethanol:water (1:1)	2.0

**Table 2:** Pure betulin obtained from 4 g of white birch bark extract.

Solvent system	Betulin (g)
Ethyl acetate:ethanol:water (4.5:4.5:1, v/v/v)	2.8
Ethyl acetate:ethanol (1:1, v/v)	2.2
Hot toluene	2.7

In order to evaluate the usefulness of the water, ethyl acetate, ethyl alcohol solvent system, the extraction from plants of compounds that have different structures was carried out. The isolation of trimyristin from nutmeg is a popular introductory-level college organic chemistry experiment. Trimyristin is routinely extracted using hot ether or dichloromethane followed by crystallization from acetone. The extraction of trimyristin using ethyl acetate, ethanol and water in the ratio of 4.5:4.5:1, at room temperature was not efficient, but at reflux, an 8.0% product recovery was achieved without using diethyl ether, dichloromethane or acetone. Crystals of trimyristin formed in the extraction solvent upon cooling, which simplified the isolation of the crude product. The product obtained after washing with a fresh, cold mixture of solvent system similar to the one used for extraction, was deemed pure and recrystallization from acetone was not necessary.

Carnosic acid has been extracted from rosemary by different methods, including supercritical carbon dioxide and super-heated water [17,18,19,20,21,22,23]. Using ethyl acetate, ethanol and water in the volume ration of 4.5:4.5:1, the amount of carnosic acid extracted was comparable to that obtained by using other solvents (Table 3).

**Table 3:** Carnosic acid from rosemary using different extraction methods.

Solvent	Carnosic acid mg/g
Ethyl acetate:ethanol:water	32.5
Acetone	26.3
Methanol	20.5

The isolation of eugenol from cloves was also accomplished without applying dichloromethane. However, like in the case of trimyristin, the extraction solvent conditions were modified. Instead of using a homogeneous mixture of ethyl acetate, ethyl alcohol and water in the volume ration of 4.5:4.5:1, the solvent system was changed. A mixture of 5% sodium chloride and ethyl acetate was used. The salting-out process enabled the product to accumulate in the organic solvent. After separating the organic layer from the aqueous mixture, followed by concentrating under vacuum, pure eugenol was obtained after using a short silica gel column. The purity was checked by thin layer chromatography.

In order to further test the efficacy of extraction at room temperature without isolating any one pure compound, four spices and white oak wood that contain antioxidants [24,25] were extracted twice, and the radical-scavenging activity of the two different extracts measured by 2,2-diphenyl-1-picrylhydrazyl (DPPH) assay [26]. The total phenolic content of each spice was also determined using the Folin–Ciocalteau assay [27]. In all cases (Table 4), the LC_50_ in mg phenol/L of the first extract was more than 20 times that of the second extract, indicating that the first extract removed more than 95% of the desired compounds.

**Table 4:** Extraction effectiveness of ethyl acetate, ethyl alcohol, and water in the volume ratio of 4.5:4.5:1 on spices.

Plant	First extraction LC_50_ (mg of phenol/L)	Second extraction LC_50_ (mg of phenol/L)
Rosemary	414.0	>9409
Sage	787.9	>16,530
Thyme	483.5	>10,626
Oregano	591.5	>12,830
White oak	879.5	>19,338

The spices used in this study contain several known antioxidative phenolic compounds with different structures, and the free-radical scavenging assay provided a good method of evaluating the efficiency of the extraction procedure before pure compounds are isolated.

## 3. Experimental Section

Organic solvents and other chemicals were purchased from Sigma Aldrich and used without purification. White oak particles were obtained from Kairos Global, LLC (Shreveport, LA, USA). Spices produced by McCormick^®^ were purchased from grocery stores in southeastern Pennsylvania. White birch bark was obtained from Ontario, Canada. All plant materials were dried in an oven at 80 °C to constant weight. Melting points were determined using a Melt-Temp apparatus (Laboratory Devices, Cambridge, MA, USA), and are not corrected. The IR spectrum was obtained using a Buck Scientific Model 500 spectrophotometer. Fourier transform NMR spectra were recorded at 400 MHz for ^1^H on a Bruker DRX 400 spectrometer in CDCl_3_ at 25 °C.

### 3.1. Extraction of Betulin from White Birch Bark

Ground white birch bark (20.0 g) in ethyl acetate, ethyl alcohol and water in 4.5:4.5:1, volume ratio (50 mL) was allowed to stand at room temperature for 96 h. Upon concentration under vacuum, a solid precipitated out of solution. Ethyl acetate was added to the mixture and the organic solvent collected and concentrated to yield a solid consisting of two major spots by TLC. Purification of betulin was performed by flash chromatography on silica gel (30–60 μm) using a mixture of hexane and ethyl acetate. Lupeol eluted first as a white solid: mp 212–214 °C (lit. [28], 213–215 °C. ^1^H-NMR, δ 4.6 (brs, 1H), 4.58 (brs, 1H, 3.30 (dd, *J* = 4.6, 12.0 Hz, 1H), 2.40–2.34 (m, 1H0, 1.96–1.90 (m, 1H), 1.70 (s, 3H), 1.67–1.49) m, 10H), 1.43–1.32 (m, 8H), 1.25–1.16 (s, 4H), 1.10–0.90 (m, 1H), 1.05 (s, 3H), 0.99 (s, 3H), 0.97 (s, 3H), 0.84 (s, 3H, 0.79 (s, 3H), 0.75 (s, 3H). Following lupeol, betulin eluted as a white solid: mp 254–255 °C (lit. [29], 253–254 °C); IR (KBr), 3400, 3080, 1644, 1449, 1382, 1010, 890; ^1^H-NMR, δ 4.67 (m, 1H, 29-H), 4.59 (m, 1H, 29-H), 3.80 (d, 1H, *J* = 10.20 Hz, 28-H), 3.35 (d, 1H, *J* = 10.20 Hz, 28-H) 3.71 (m, 1H, 3-H), 2.38 (m, 1H, 19-H), 1.05–2.05 (complex, CH_2_ and CH), 1.02, 0.97, 0.97, 0.82, 0.76 (all s, 15H, 5xCH_3_).

### 3.2. Extraction of Carnosic Acid from Rosemary and Analysis by HPLC

Finely ground rosemary leaves (10.0 g) was placed in 100 mL of ethyl acetate, ethyl alcohol and water in 4.5:4.5:1 volume ratio. After standing at room temperature for 96 h, the mixture was filtered, and a small amount of the total liquid extract analyzed by HPLC using an Aqua C-18–125A (150 × 4.0 mm, 5 micron) from Phenomenex (Torrance, CA, USA). The elution was accomplished with a gradient starting at 10% methanol and 90% water containing acetic acid (2.5%).

### 3.3. Extraction of Trimyristin

Nutmeg (2.0 g) and three boiling chips were added to 25 mL of ethyl acetate, ethanol and water in 4.5:4.5:1 volume ratio. The mixture was refluxed gently with occasional shaking. There was minimum bumping during heating. After one hour, the mixture was vacuum filtered while still hot, and the hot solution quickly transferred to a 25 mL beaker and left at room temperature for the crystals to form. The cold mixture was vacuum filtered, crystals washed with cold ethyl acetate, ethanol and water in 4.5:4.5:1, volume ratio (5 mL) and dried to obtain 0.16 g, 8.0% recovery, mp 56–58 °C.

### 3.4. Extraction of Eugenol from Cloves

Coarsely ground dry cloves (4.95 g) in a mixture of ethyl acetate (30 mL) and 5% sodium chloride solution (10 mL) were placed in 100 mL flask and heated at reflux for 1 hour. The hot mixture was filtered; the residue washed with ethyl acetate (20 mL), and the filtrate placed in a 100 mL separatory funnel to remove the aqueous layer. After collecting the organic layer, the aqueous layer was back extracted with 10 mL of ethyl acetate. The combined organic extract was washed with 10 mL of water and concentrated under vacuum. The resulting oil was dissolved in ethyl acetate (10 mL) and filtered to remove solid particles. After concentration 0.46 g of oil was recovered. The product was purified on a short silica gel column using hexane and ethyl acetate as eluent. The extracted product was found to be pure by thin layer chromatography using commercial eugenol as a standard.

### 3.5. Extraction of Plant Material for Determining Free-Radical Scavenging Activity and Total Phenolic Content

Crushed plant material (1.0 g) was dissolved in ethyl acetate, ethyl alcohol and water in 4.5:4.5:1 volume ratio (50 mL). After standing at room temperature for 96 h, the mixture was filtered. The residue was washed with 10 mL of extracting solvent and filtered. The two solutions were combined and aliquots were used for the DPPH and total phenolic content assays without concentration. The residue was then dried and re-extracted with fresh ethyl acetate, ethyl alcohol and water in 4.5:4.5:1 volume ratio (50 mL). After 96 h at room temperature the product was treated as in the first extraction before carrying out the DPPH and the total phenol assays to compare antioxidative activity.

### 3.6. Determination of the Free-Radical Scavenging Activity

The free-radical scavenging activity was measured by the 2,2-diphenyl-1-picrylhydrazyl (DPPH) method described by Blois, with some modification [17]. Different amounts of extract and methanol were added to a solution of 0.3 mg/mL methanolic solution of DPPH to make up a total volume of 3.0 mL. After standing for 30 min at room temperature, the absorbance was measured at 517 nm using an SQ-2800 UV/Visible-Cole Palmer Instruments Company (Vernon Hill, IL, USA). High absorbance of the reaction mixture indicated low free radical scavenging activity. The IC_50_ value, defined as the amount of antioxidant necessary to decrease the initial DPPH concentration by 50%, was estimated from the results using Microsoft Excel. The capability to scavenge the DPPH radical was calculated using the following equation:


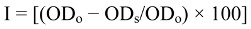


where I was the inhibition percentage; OD_o_, was the absorbance of the negative control (containing 100 μL of MeOH instead of the sample); and OD_s_ was the absorbance of the samples. The experiment was carried out in triplicate and the results are average values.

### 3.7. Determination of Total Phenol

Determination of the amount of phenol as gallic acid equivalent was carried out using Folin-Ciocalteau method with minor modifications. The spice extract (20 μL), water (1.58 mL), Folin-Ciocalteau reagent (100 μL) and saturated sodium carbonate solution (300 μL) were incubated at 40 °C. After 30 min, optical density was read at 765 nm, and the amount of total phenol calculated using a calibration curve.

## 4. Conclusions

A mixture of ethyl acetate, ethyl alcohol and water in the volume ration of 4.5:4.5:1 was effective in extracting betulin from white birch at room temperature. The amount recovered was similar to that obtained using other solvents like chloroform, dichloromethane and toluene. The solvent system was also efficient in extracting antioxidants from spices. Additionally, eugenol and trimyristin were extracted using a modified solvent system at high temperature. Overall, the relatively eco-friendly solvent system was capable of extracting compounds with different structures at low temperature. This indicates that the system can be used to extract other compounds provided those compounds are stable under the experimental conditions.
